# Hydrocephalus in cerebral venous thrombosis

**DOI:** 10.1007/s00415-015-7652-4

**Published:** 2015-02-07

**Authors:** Susanna M. Zuurbier, René van den Berg, Dirk Troost, Charles B. Majoie, Jan Stam, Jonathan M. Coutinho

**Affiliations:** 1 Department of Neurology, Academic Medical Centre, University of Amsterdam, Meibergdreef 9, Amsterdam, 1105 AZ The Netherlands; 2 Department of Radiology, Academic Medical Centre, University of Amsterdam, Amsterdam, The Netherlands; 3 Department of Pathology, Academic Medical Centre, University of Amsterdam, Amsterdam, The Netherlands

**Keywords:** Cerebral venous thrombosis, Sinus thrombosis, Imaging, Hydrocephalus

## Abstract

Increased intracranial pressure is common in cerebral venous thrombosis (CVT), but hydrocephalus is rarely reported in these patients. We examined the frequency, pathophysiology and associated clinical manifestations of hydrocephalus in patients with CVT admitted to our hospital between 2000 and 2010 (prospectively since July 2006). Hydrocephalus was defined as a bicaudate index larger than the 95th percentile for age, and/or a radial width of the temporal horn of ≥5 mm. We excluded patients in whom hydrocephalus was caused by a disease other than CVT or if it was iatrogenic. 20 out of 99 patients with CVT had hydrocephalus. 6 patients with hydrocephalus were excluded from the analysis. Patients with hydrocephalus more often had focal neurological deficits (86 vs. 49 %, *p* = 0.02) and were more frequently comatose (43 vs. 16 %, *p* = 0.06), as compared to patients without hydrocephalus. Deep cerebral venous thrombosis (64 vs. 9 %, *p* < 0.001) and edema of the basal ganglia and thalami (64 vs. 4 %, *p* < 0.001) were more common in patients with hydrocephalus. Intraventricular hemorrhage was present in 1 patient with hydrocephalus, compared to none among patients without hydrocephalus (7 vs. 0 %, *p* = 0.15). Outcome at follow-up was worse in patients with hydrocephalus (mRS 0–1, 36 vs. 68 %, *p* = 0.02; mortality 29 vs. 9 %, *p* = 0.07). Hydrocephalus occurs more frequently in cerebral venous thrombosis than previously believed, especially in patients with deep cerebral venous thrombosis and edema of the basal ganglia. The presence of hydrocephalus is associated with a worse clinical outcome, but a direct causal relation is unlikely. Routine shunting procedures are not advisable.

## Introduction

Cerebral venous thrombosis (CVT) is a rare form of stroke with an estimated incidence of 1.3 per 100.000 among adults [1]. Thrombosis of the sinuses leads to impairment of cerebral spinal fluid drainage and venous outflow, which often causes increased intracranial pressure, with symptoms such as headache, papilledema and 6th nerve palsy [2–4]. Hydrocephalus, however, is rarely reported in patients with CVT and few studies have investigated this complication. Cohort studies of CVT reported incidences of hydrocephalus between 0.2 and 6.6 % [5–7]. However, hydrocephalus was not a major research topic in any of these studies and specific definitions or details are not provided. All studies also lacked central review of imaging data and the validity of the estimates provided by these studies is, therefore, questionable. In fact, the only detailed reports on hydrocephalus in patients with CVT are case reports [8–12]. The aim of the present study was to systematically examine the incidence, risk factors, pathophysiology, associated clinical manifestations, and outcome of hydrocephalus in a large cohort of consecutive patients with CVT.

## Materials and methods

### Study population

We included consecutive patients with CVT aged 12 and older admitted to the Academic Medical Centre (University of Amsterdam) between January 1st 2000 and January 1st 2011. Our hospital serves as a tertiary referral center for CVT cases in the Netherlands. Since July 2006, all patients with CVT are enrolled in a prospective database, as described previously [13]. We retrospectively identified patients admitted between January 1st 2000 and June 30th 2006, using the International Classification of Diseases, 9th revision and the Dutch financial coding system for hospital care [1]. CVT was confirmed in all patients by one of the following—magnetic resonance imaging (MRI) with MRI-venography, computed tomographic-venography (CT-V), conventional angiography, or autopsy. We collected data on demographics, baseline clinical characteristics, and treatment. Clinical outcome was classified according to the modified Rankin Scale (mRS), a 7-point scale which ranges from 0 (complete recovery) to 6 (dead). We defined good outcome as a mRS score of 0 or 1 at last follow-up. Patients admitted before July 2006 for whom no mRS had been recorded were contacted by telephone to determine the score by means of a structured interview [14]. Under Dutch law, ethical approval did not have to be obtained for this observational study.

### Imaging data and definition of hydrocephalus

All cerebral imaging results were re-evaluated by two neuroradiologists (RvdB and CBM). The type of scan, location of the thrombosis, presence and type of intracranial lesions, and the presence of hydrocephalus were documented. We determined the presence of hydrocephalus by measuring the bicaudate index (BCI) and the radial width of the temporal horn (rWTH), as described previously [15, 16]. Briefly, the BCI is the width of the frontal horns at the level of the caudate nuclei and the foramen of Monro divided by the corresponding width of the brain at the same level. To calculate age-adjusted relative sizes, the BCIs were divided by the corresponding upper limit (95th percentile) per age group, as previously reported [16].

The rWTH of the lateral ventricle was measured at the tip of the temporal horn on (axial) imaging using the method described by Frisoni et al. [15]. The rWTH was measured at the image slice where the temporal horn was the widest. Two parallel lines were drawn tangential to the margins of the temporal horn at its widest point. The rWTH is the distance between the two lines. Because control values for the rWTH have not been published, we measured the rWTH in a control group of healthy adults. For each patient with CVT, we selected an age-matched control patient who underwent cranial CT imaging because of a minor head injury between 2010 and 2012. Only patients without neurological co-morbidity and in whom the CT scan was unremarkable (without any traumatic injuries) were eligible as controls.

We defined hydrocephalus as one of the following—a BCI above the 95th percentile for age, and/or a rWTH (uni- or bilateral) 2.5 standard deviations or more above the mean rWTH of the control group. Presence of hydrocephalus was determined on baseline imaging and on follow-up scans performed within 30 days of diagnosis. If a patient had hydrocephalus on multiple examinations, we used the scan in which the hydrocephalus was most severe. We excluded patients in whom hydrocephalus was caused by a disease other than CVT or if it was deemed iatrogenic (e.g. following decompressive hemicraniectomy).

### Statistical analysis

For categorical variables, we used the Pearson *χ* ^2^ or Fisher’s exact test if appropriate. The Student’s *t* test or Mann–Whitney test (for skewed distributions) were used for continuous variables. To determine whether hydrocephalus was associated with outcome, we performed multivariate logistic regression analysis. All analyses were performed with SPSS software, version 19.0.

## Results

The mean rWTH in the age-matched control group (no CVT) was 1.8 mm with a standard deviation of 1.2 mm. Therefore, the upper limit of normal for the rWTH was defined as 5 mm.

**Fig. 1:**
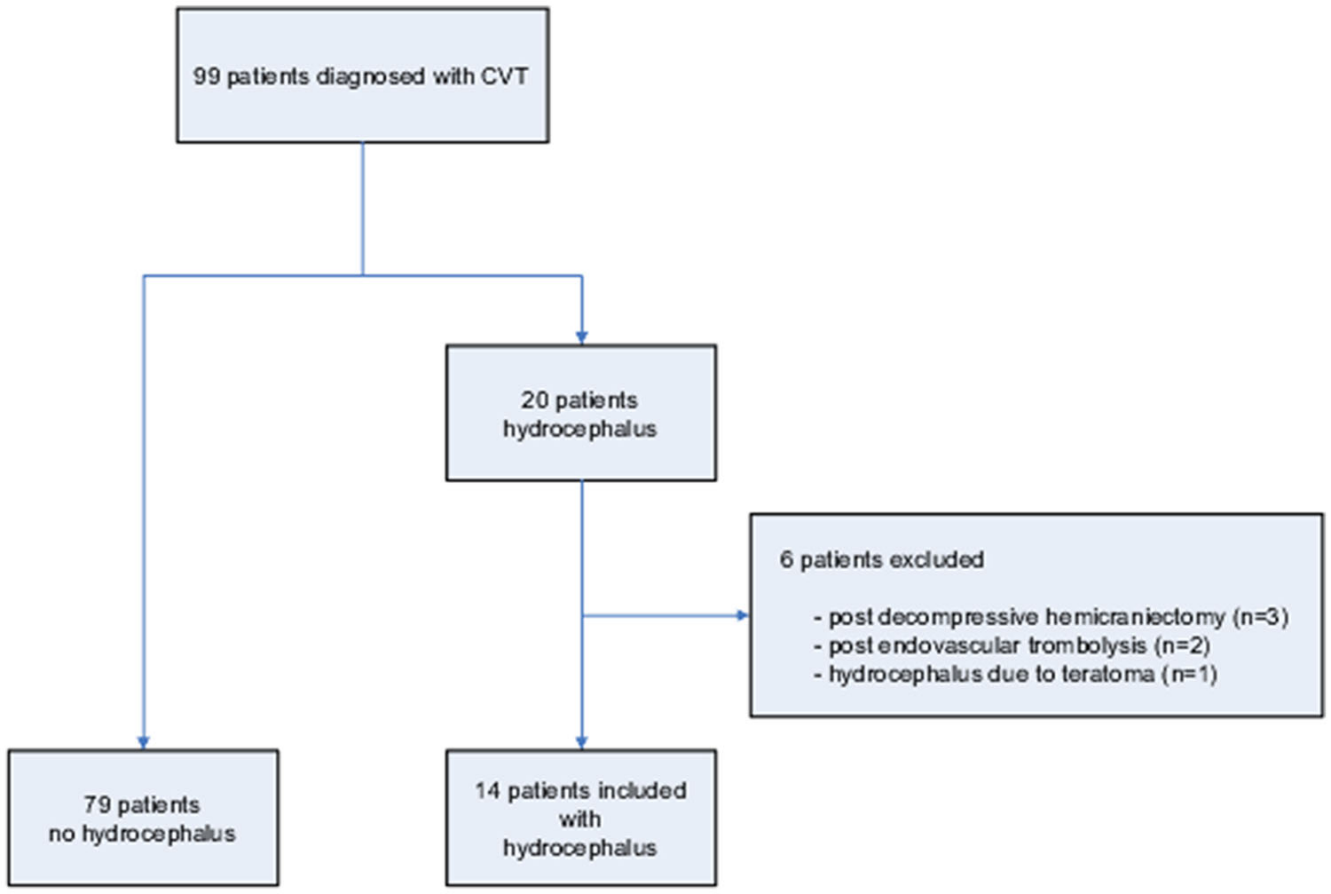
Flowchart of patient selection

During the study period, 99 patients with CVT older than 12 were admitted, of whom 59 after July 2006 (prospective cohort). Twenty of these patients (20 %) had hydrocephalus (Fig. 1). Six patients were excluded from the analysis because the hydrocephalus was iatrogenic or had another cause [after decompressive hemicraniectomy (3); complication of endovascular thrombolysis (2) and hydrocephalus due to a teratoma (1)]. Therefore, the study cohort consisted of 14 patients with and 79 patients without hydrocephalus. In 10 of 14 patients (71 %) hydrocephalus was present at baseline. In the remaining patients, hydrocephalus was found within 24 h of diagnosis (3 patients) or 8 days after diagnosis (1 patient). Thirteen patients (93 %) had an increased rWTH (bilateral in 8) and only 2 (14 %) had an increased BCI (Table 1). One patient had both an increased BCI and rWTH. The mean rWTH was 6.5 mm in the patients with hydrocephalus, compared to 2.1 mm in those without hydrocephalus. At baseline, patients with hydrocephalus more often had focal neurological deficits (86 vs. 49 %, *p* = 0.02), fixed and dilated pupil(s) (14 vs. 1 %, *p* = 0.06), and a lower Glasgow Coma Scale (median 10 vs. 15, *p* = 0.01) as compared to patients without hydrocephalus. Other baseline clinical characteristics did not differ significantly. Lumbar puncture was performed in four patients with hydrocephalus, and all had an increased cerebrospinal fluid pressure (>20 cm H_2_O). In patients without hydrocephalus, ten patients underwent lumbar puncture and eight had an increased pressure.

**Table 1:** Baseline characteristics

	Hydrocephalus (*n* = 14)	No hydrocephalus (*n* = 79)	*P* value
Hydrocephalus details			
Increased rWTH	13/14 (93 %)	–	NA
Bilateral	8/14 (57 %)	–	NA
Mean rWTH (mm, SD)	6.5 (1.8)	2.1 (1.4)	NA
Increased BCI	2/14 (14 %)	–	NA
Mean BCI (SD)	0.13 (0.04)	0.11 (0.03)	NA
Hydrocephalus at baseline	10/14 (71 %)	–	NA
Demographics			
Female	13/14 (93 %)	53/79 (67 %)	0.06
Mean age (SD)	33 (16)	37 (13)	>0.1
Symptoms and signs			
Duration symptom onset to diagnosis (days, median, IQR)	5 (2–12)	4 (2–7)	>0.1
Duration admission to diagnosis (days, median, IQR)	0 (0–1)	1 (0–2)	>0.1
Headache	13/14 (93 %)	67/79 (85 %)	>0.1
Focal neurological deficit	12/14 (86 %)	38/77 (49 %)	0.02
Seizure(s)	3/14 (21 %)	27/79 (34 %)	>0.1
Glasgow coma scale (median, IQR)	10 (8–14)	15 (11–15)	0.01
Coma	6/14 (43 %)	12/73 (16 %)	0.06
Fixed and dilated pupil(s)	2/14 (14 %)	1/79 (1 %)	0.06

*rWTH* radial width of the temporal horn, *BCI* bicaudate index, *SD* standard deviation, *IQR* interquartile range

Patients with hydrocephalus more often had thrombosis of the straight sinus (64 vs. 23 %, *p* = 0.002) and deep cerebral venous thrombosis (DCVT: internal cerebral veins, vein of Galen, and/or the basal vein of Rosenthal; 64 vs. 9 %, *p* < 0.001, Table 2). In contrast, the superior sagittal sinus was less often involved in patients with hydrocephalus (21 vs. 67 %, *p* = 0.001). Hypodensity of the basal ganglia and/or thalami on CT, suggesting edema, was present in 9/14 (64 %) patients with hydrocephalus, as compared to 3/79 (4 %) in those without hydrocephalus (*p* < 0.001). In two of these nine patients, the presence of edema was confirmed with MRI. There was no difference in frequency of baseline intracerebral hemorrhages between the two groups (50 vs. 54 %). One patient with hydrocephalus had both supra and infratentorial localization of the hemorrhage. Intraventricular extension of the hemorrhage was present in one patient with hydrocephalus at baseline, compared to none among patients without hydrocephalus (7 vs. 0 %, *p* = 0.15). This patient had a small amount of blood in the right occipital horn of the lateral ventricle.

**Table 2:** Radiological findings, treatment and outcome

	Hydrocephalus (*n* = 14)	No hydrocephalus (*n* = 79)	*P* value
Thrombosed sinuses			
Superior sagittal sinus	3/14 (21 %)	53/79 (67 %)	0.001
Lateral sinus (left and/or right)	12/14 (86 %)	59/79 (75 %)	>0.1
Straight sinus	9/14 (64 %)	18/78 (23 %)	0.002
Deep cerebral venous system^a^	9/14 (64 %)	7/79 (9 %)	<0.001
Thrombosis >1 sinus	13/14 (93 %)	67/79 (85 %)	>0.1
Parenchymal lesions			
Edema basal ganglia/thalami^b^	9/14 (64 %)	3/79 (4 %)	<0.001
Intracerebral hemorrhagic lesion	7/14 (50 %)	43/79 (54 %)	>0.1
Intraventricular hemorrhage	1/14 (7 %)	0/79 (0 %)	0.15
Treatment			
Heparin treatment	13/14 (93 %)	79/79 (100 %)	>0.1
Endovascular treatment	9/14 (64 %)	14/78 (18 %)	<0.001
Decompressive hemicraniectomy	3/14 (21 %)	6/78 (8 %)	>0.1
Ventricular shunting procedure	1/14 (7 %)	2/79 (3 %)	>0.1
Clinical outcome at last follow-up			
Duration of follow-up (median months, IQR)	16 (2–75)	8 (4–24)	>0.1
mRS 0–1 (recovery without handicap)	5/14 (36 %)	50/74 (68 %)	0.02
Mortality at follow-up	4/14 (29 %)	7/75 (9 %)	0.07

*IQR* interquartile range, *mRS* modified Rankin Scale

^a^Deep cerebral venous system was defined as thrombosis in one or more of the following veins: internal cerebral veins, vein of Galen, and basal vein of Rosenthal

^b^Confirmed by MRI in 2/9 patients with hydrocephalus

**Fig. 2:**
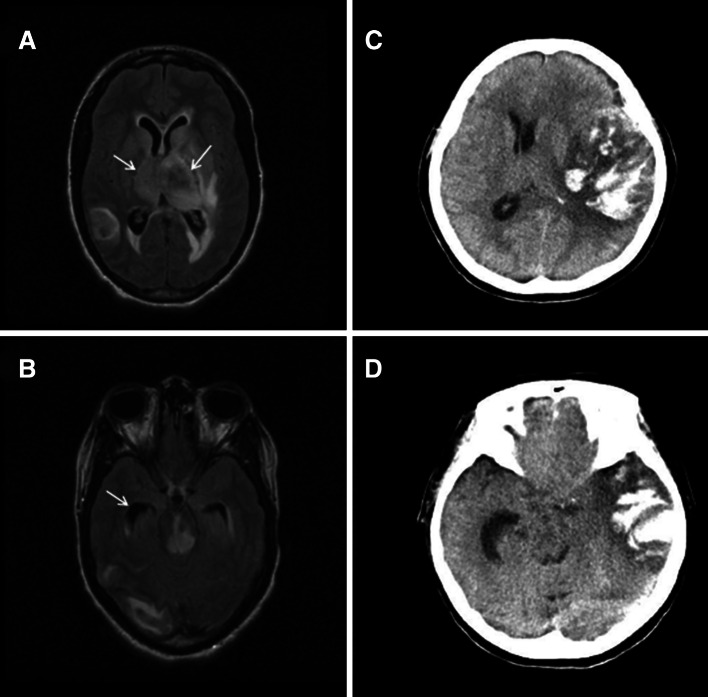
Cerebral imaging of two patients with hydrocephalus. **a**, **b** Axial Fluid Attenuated Inversion Recovery (FLAIR) MRI showing extensive edema in the thalami, basal ganglia and brainstem, and hydrocephalus of both temporal horns (*arrows*). **c**, **d** Axial non-contrast-enhanced CT scan of a different patient showing a large space occupying intracranial hemorrhage in the left hemisphere, and an increase of width of the contralateral temporal horn

Three different patterns of hydrocephalus could be discerned. The largest group consisted of patients with an increased rWTH, deep cerebral venous thrombosis and edema of the basal ganglia and thalami (Fig. 2a, b). Nine of the 14 patients with hydrocephalus fitted this pattern. The BCI was normal in all of these patients, and only one had thrombosis of the superior sagittal sinus. The next group consisted of three patients with an increased unilateral rWTH due to mass effect from a contralateral intracerebral hemorrhage (Fig. 2c, d). Finally, two patients had an increased BCI without parenchymal lesions or thrombosis of the deep venous system. Both these patients had superior sagittal sinus thrombosis, without involvement of the deep veins.

**Fig. 3:**
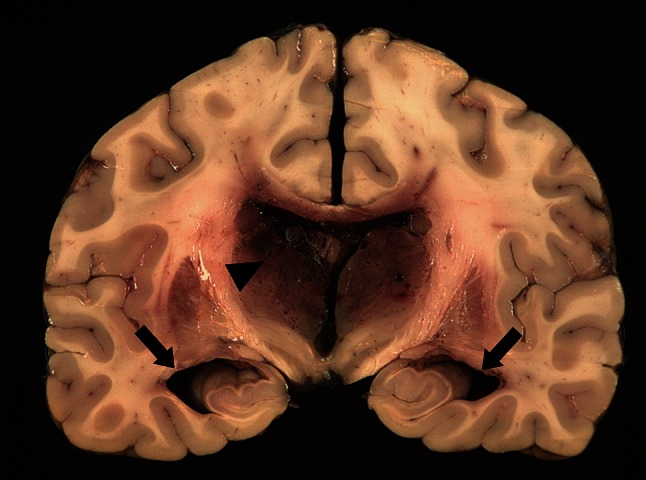
Autopsy of a patient with hydrocephalus. Coronal section of the brain showing enlargement of the temporal horns (*arrows*). Small petechial hemorrhages and infarcts are present bilaterally in the thalami (*arrowhead*). The third ventricle and foramen of Monro are narrowed

Autopsy was performed in one patient with hydrocephalus and DCVT. Figure 3 shows a coronal section of the brain demonstrating widening of the temporal horns and small petechial hemorrhages and infarcts in the thalami. The third ventricle and foramen of Monro were narrowed.

All patients except one (in whom CVT was not diagnosed until autopsy) received treatment with therapeutic doses of heparin (Table 2). Patients with hydrocephalus more often were treated with endovascular thrombolysis (64 vs. 18 %, *p* < 0.001) and more often underwent decompressive hemicraniectomy (21 vs. 8 %, *p* > 0.1). One patient with hydrocephalus received an external ventricular drain, which was done prior to the diagnosis of CVT. This shunting procedure was done because of clinical worsening (decrease in consciousness) and for diagnostic reasons. At that time bacterial meningitis was considered the most likely diagnosis and previous attempts to obtain cerebrospinal fluid through lumbar puncture had failed. She suffered from acute lymphoblastic leukemia, which was diagnosed several hours after placement of the drain and died as a result of a massive intraventricular hemorrhage 1 day after admission. No shunting procedures were performed in any of the other patients with hydrocephalus. Two patients without hydrocephalus received a ventricular peritoneal drain because of intracranial hypertension with severe papilledema and impending blindness. Both patients recovered without visual loss.

Follow-up information on clinical outcome was available for 88 of the 93 patients (95 %). While the duration of follow-up was non-significantly longer for patients with hydrocephalus (16 vs. 8 months, *p* > 0.1), patients with hydrocephalus less often had a good outcome at last available follow-up (36 vs. 68 %, *p* = 0.02). Mortality was also higher in patients with hydrocephalus (29 vs. 9 %, *p* = 0.07). After adjustment for baseline clinical imbalances (gender, focal neurological deficits, coma, and fixed pupils), however, hydrocephalus was not an independent predictor of outcome (adjusted OR for mRS 0–1: 0.67, 95 % CI 0.15–2.92).

## Discussion

This is the first study on hydrocephalus in a large cohort of consecutive patients with cerebral venous thrombosis. Our data show that hydrocephalus predominantly occurs in patients with deep cerebral venous thrombosis and edema of the basal ganglia and thalami. In all patients with this pattern, the hydrocephalus is limited to the temporal horns of the lateral ventricle. It is unlikely that hydrocephalus in these patients is due to a diminished resorption of cerebrospinal fluid by the arachnoid villi. The majority of these villi are located in the superior sagittal sinus, [17] which was rarely occluded in these patients. A more plausible explanation and also consistent with the pattern of hydrocephalus, is that the flow of cerebrospinal fluid (CSF) through the foramen of Monro is obstructed due to local compression by edema of the basal ganglia and/or thalami. The autopsy results are in agreement with this hypothesis. Considering the pivotal role of the arachnoid villi in the drainage of CSF, one may wonder why CVT does not result in hydrocephalus more often, especially in cases with thrombosis of the superior sagittal sinus. Many of these patients have increased intracranial pressure, but hydrocephalus occurred only in 2/55 patients with thrombosis of the superior sagittal sinus in our cohort. The most likely explanation is that there is no pressure gradient of the CSF in these cases between the ventricular and subarachnoid compartments at the cerebral convexity. A similar mechanism has been proposed in patients with cryptococcal meningitis and increased intracranial pressure, who generally also do not have hydrocephalus [18]. Intraventricular extension of hemorrhage may also be a contributing factor to the development of hydrocephalus, but this occurred only in one patient.

The frequency of hydrocephalus was much higher in our cohort than previously reported [5–12]. There are two possible explanations for this disparity. First, our hospital is a tertiary referral center for patients with a severe form of CVT. 21 % of our patients was in coma at baseline and 17 % had DCVT, which is both higher than in other cohort studies [2, 19]. A second explanation is that most studies did not focus on hydrocephalus, which probably leads to an underestimation of its frequency.

Despite aggressive treatment with endovascular thrombolysis and decompressive hemicraniectomy in many patients, hydrocephalus was associated with a high risk of poor outcome. After correction for baseline imbalances, however, hydrocephalus was not an independent predictor of outcome. Hydrocephalus is probably a marker of a severe form of CVT, associated with edema of the basal ganglia/thalami caused by DCVT. DCVT is a well-known predictor of poor outcome [2]. Since a causal relation between hydrocephalus and poor outcome is unlikely, the risks of shunting procedures and the fact that patients with CVT require treatment with anticoagulation, we do not recommend routine shunting procedures in patients with CVT and hydrocephalus. Generally, we will only consider an external ventricular drain in these patients if they are in a worse clinical condition than would be expected on the extent of the parenchymal lesions or if they deteriorate without apparent cause other than the hydrocephalus. In patients who additionally have seizures, the decision whether or not to perform a shunting procedure is even more difficult, since these patients often have fluctuations in their consciousness. The other situation when a shunting procedure should be considered in CVT is in patients with severe intracranial hypertension (usually without hydrocephalus) that comprises visual function or, rarely, cerebral perfusion.

Our study has some limitations—first, the rWTH is less often used for the determination of hydrocephalus than the BCI. Since cutoff values for a normal rWTH were not available in the literature, we determined the upper normal value by measuring the rWTH in a cohort of age-matched patients. Another limitation is that MRI—which is clearly superior to determine the extent of edema of the basal ganglia—was only available in a minority of patients. Finally, in a subset of patients, the data were collected retrospectively. However, since all imaging data were re-evaluated and outcome was available for almost all patients, we do not think this influences the validity of the results.

In conclusion, we found that hydrocephalus is more common in patients with cerebral venous thrombosis than previously believed. Hydrocephalus mainly occurs in patients with deep cerebral venous thrombosis and edema of the basal ganglia and thalami, probably because of obstruction of the foramen of Monro. The presence of hydrocephalus is associated with a worse clinical outcome, but a direct causal relation is unlikely. Routine shunting procedures are, therefore, not recommended in these patients.
